# Low Attainment of Treatment Targets for Cardiovascular Risk Factors in Indonesian Adults With Established Coronary Artery Disease

**DOI:** 10.1111/jep.14311

**Published:** 2025-01-13

**Authors:** Dian Sidik Arsyad, Nurul Qalby, Andriany Qanitha, Idar Mappangara, Wilis Milayanti, Wahiduddin, Ansariadi, Frank L. J. Visseren, Maarten J. Cramer, Pieter A. Doevendans, Steven H. J. Hageman

**Affiliations:** ^1^ Division of Heart and Lungs, Department of Cardiology, University Medical Center Utrecht University of Utrecht Utrecht the Netherlands; ^2^ Department of Epidemiology, Faculty of Public Health Hasanuddin University Makassar Indonesia; ^3^ Department of Public Health and Family Medicine, Faculty of Medicine Hasanuddin University Makassar Indonesia; ^4^ Department of Physiology, Faculty of Medicine Hasanuddin University Makassar Indonesia; ^5^ Department of Cardiology and Vascular Medicine, Faculty of Medicine Hasanuddin University Makassar Indonesia; ^6^ Department of Vascular Medicine University Medical Center Utrecht Utrecht the Netherlands; ^7^ Netherlands Heart Institute Utrecht the Netherlands

**Keywords:** cardiovascular disease, coronary artery disease, epidemiology, risk factor control, secondary prevention, treatment target

## Abstract

**Rationale:**

Established coronary artery disease (CAD) patients are at increased risk for recurrence of cardiovascular events and mortality due to non‐attainment of recommended risk factor control targets.

**Objective:**

We aimed to evaluate the attainment of treatment targets for risk factor control among CAD patients as recommended in the Indonesian CVD prevention guidelines.

**Methods:**

Patients were consecutively recruited from the Makassar Cardiac Center at Wahidin Sudirohusodo Hospital, Indonesia. Targets for systolic blood pressure (SBP < 140 mmHg), low‐density lipoprotein‐cholesterol (LDL‐C < 1.8 mmol/L), body mass index (BMI: 20–25 kg/m^2^), non‐smoking status and antithrombotic use were assessed according to the national guideline. Attainment levels were evaluated in CAD population subgroups using logistic regression to identify patients who required more intensive monitoring.

**Results:**

A total of 395 CAD patients (median age: 57 years (IQR: 49–65), 63.8% men) were recruited between February and June 2021. We observed that 1.8% of the CAD patients met all risk factor treatment targets. LDL‐C had the lowest attainment level (5.1%), followed by BMI (59.7%), SBP (62.8%), antithrombotic usage (76.7%) and non‐smoking status (94.4%). Apart from lower attainment of SBP control in the 50+ years age group (aOR: 0.57, 95% CI: 0.35–0.94) and women (aOR: 0.53, 95% CI: 0.34–0.83), the attainment levels of other risk factors were consistently low across age, sex, marital status and educational subgroups.

**Conclusion:**

These findings emphasize the urgent need for effective management and heightened awareness, particularly for controlling LDL‐C in the CAD population. Action to address this issue is crucial for mitigating the CVD burden, particularly in low‐ and middle‐income countries such as Indonesia.

## 1 Introduction

Cardiovascular diseases (CVDs) continue to be the leading cause of morbidity and mortality globally, with an escalating prevalence of uncontrolled CVD risk factors, especially in developing countries [1, 2]. For instance, in a previous study, Indonesia showed that approximately 65% of individuals with established CVD have a very high 10‐year risk of recurrent events and mortality [3]. Among CVD cases, coronary artery disease (CAD) is the most common and foremost single cause of mortality and loss of disability‐adjusted life years (DALYs) worldwide [4].

The 2022 Indonesian guidelines for the prevention of atherosclerotic cardiovascular disease (ASCVD) incorporate recommendations from the American College of Cardiology/American Heart Association and the European Society of Cardiology to establish explicit treatment goals for both primary and secondary prevention, with a strong emphasis on managing traditional risk factors [5, 6, 7]. The Indonesian Heart Association guidelines for secondary prevention in patients with CAD adopt a comprehensive strategy that includes lifestyle modifications such as smoking cessation, adherence to a heart‐healthy diet, and regular physical activity. Pharmacological interventions are centred around lipid management, with a target low‐density lipoprotein (LDL) level of < 70 mg/dL, blood pressure control aiming for a threshold below 130/80 mmHg, and glycemic control for diabetic patients with an HbA1c target of less than 7%. Additionally, antithrombotic therapy with aspirin or P2Y12 inhibitors, beta‐blockers, ACE inhibitors, or ARBs is recommended for specific clinical profiles, such as those with a history of myocardial infarction (MI), heart failure or hypertension. The guidelines also emphasize weight management to achieve a normal BMI and address psychosocial factors like stress and depression, all of which are critical to reducing recurrent cardiovascular events and improving long‐term outcomes [7].

In many low‐ and middle‐income countries (LMICs) within the Southeast Asia region, adherence to cardiovascular guidelines and protocols remains low. Disease management and clinical decision‐making are still predominantly influenced by the authority of medical doctors [8]. In our previous study, we highlighted the barriers and key challenges faced by cardiovascular health services in LMICs, particularly in Indonesia. These challenges stem from issues within the healthcare system (such as limited access to care, inequality, geographical barriers, unavailable or unaffordable cardiovascular services in primary care, and insufficient capacity to diagnose, monitor, and manage CVDs, including hypertension, diabetes, and dyslipidemia); healthcare providers (including the limited availability of healthcare professionals, lack of standardization among providers, authority in decision‐making, and poor post‐discharge care management); and patient‐related factors (including low awareness of cardiovascular symptoms, financial constraints, poor adherence to medications, and low levels of education regarding guideline recommendations) [8]. Our previous study demonstrated that only 44.1% of CAD patients adhered to their prescribed medications at a 30‐day follow‐up, and poor medication adherence was an independent predictor of post‐discharge mortality, irrespective of the underlying CAD diagnosis [9].

Despite robust evidence supporting the effectiveness of preventive interventions, a significant number of patients struggle to achieve the recommended levels for key CVD risk factors, even in developed countries [10, 11, 12, 13]. The failure to attain optimal risk factor control contributes to an increased incidence of recurrent events and mortality within these populations and potentially plays a vital role in the exceptionally high risk of recurrent events among Indonesian CAD patients [3].

Our study aimed to assess the attainment of risk factor treatment targets among Indonesian patients with established CAD following the 2022 Indonesian CVD prevention guidelines. Additionally, we sought to identify patient subgroups with lower attainment of the recommended treatment targets.

## 2 Methods

### 2.1 Study Design and Population

This cross‐sectional study was conducted on patients with established CAD at the Makassar Cardiac Center (MCC) in Dr. Wahidin Sudirohusodo Hospital, South Sulawesi, Indonesia. With a population of 1.5 million people, Makassar is the largest city in the eastern part of Indonesia and the fifth most populous city in the country. The MCC serves as the primary cardiac referral centre for the East Indonesian region and mainly delivers cardiovascular‐related health services to the population of South Sulawesi and its surrounding areas.

Patients visiting the outpatient clinic of the MCC were invited to participate in the study. We employed consecutive sampling by inviting every patient who met the inclusion criteria during the data collection periods (between March and June 2021). The inclusion criteria were as follows: adult participants (> 30 years of age) with a documented diagnosis of CAD, including stable angina, unstable angina and MI, or who had been treated with percutaneous coronary intervention (PCI) or coronary artery bypass graft surgery. Eligible patients who could communicate fluently in Bahasa and were willing to participate in the study were included.

The sample size for our study was estimated using a single‐proportion formula, with a 5% margin of error, a 95% confidence level, and an assumed proportion of treatment target achievement set at 50%. This proportion was chosen as it yields the highest minimum sample size compared to lower or higher values. Based on the formula, 384 patients were required. To account for potential non‐response or refusals, we increased the sample size by 10%, resulting in a target of 423 patients. During the data collection period, we approached 420 patients, of whom 395 agreed to participate in the study.

### 2.2 Data Collection

Data analyzed in the study were based on two methods of data collection. First, we used a questionnaire for collecting information regarding patient characteristics, and second, we extracted the patient's medical records for data regarding their risk factors. The questionnaire was based on standard questions for acquiring patients' demographic characteristics, including age, sex, marital status and education level (highest level completed). We also gathered information regarding self‐reported smoking status (i.e. currently smoking, quit smoking or never smoke), and the use of any antithrombotic agents (whether they were taking any anti‐platelet or any anti‐coagulant medication). Furthermore, data on CAD risk factors including systolic blood pressure (SBP), low‐density lipoprotein cholesterol (LDL‐C) and body mass index (BMI), were measured during their current visit to the MCC. The measurement and testing of patients' risk factor status were conducted according to the standardized procedures at the MCC hospitals. After patients were measured and tested for risk factors status, their data entered into the electronic medical records, we then extracted these data from the hospital medical records database.

### 2.3 Risk Factor Treatment Targets

The target values for risk factor control in the present study were defined based on the 2022 Indonesian National Guideline for ASCVD Prevention as follows: SBP < 140 mmHg, LDL‐C < 1.8 mmol/L (< 70 mg/dL), BMI: 20–25 kg/m^2^, smoking cessation or not smoking and use of antithrombotic agents (use of antiplatelet and/or anticoagulant drugs) [7].

### 2.4 Statistical Analysis

The statistical analysis was conducted using IBM SPSS Statistics for Windows, version 29.0 (IBM Corp., Armonk, NY, USA). Categorical variables are presented as percentages, while continuous variables are expressed as medians and interquartile ranges (IQRs). Factors associated with patients attaining target levels for each risk factor (i.e., SBP, LDL‐C level, BMI, smoking status and use of antithrombotic agents) were analyzed using multivariate logistic regression adjusted for all patient characteristics, including age group, sex, marital status and education level.

### 2.5 Ethical Approval

The study received ethical approval from the Ethical Review Board (ERB) of the Public Health Faculty, Hasanuddin University, Makassar, Indonesia, with document number 8361/UN4.14.1/TP.02.02/2020. All eligible participants provided and signed written informed consent before their interview.

## 3 Results

### 3.1 Patient Characteristics and Risk Factor Prevalence

The study included a total of 395 participants, with a median age of 57 years (IQR: 49–65 years), and the majority (64%) were male. Of all the participants, 24% had a positive family history of CVD before 55 years of age. On average, participants were first diagnosed with or experienced CVD events at the age of 53 years (IQR: 45–61 years). The detailed participant characteristics, stratified by sex, are presented in Table 1.

**Table 1:** Participant characteristics stratified by sex.

Patients' characteristics	Male (*n* = 252)	Female (*n* = 143)	Total (*n* = 395)
Age; median (IQR) (in years)	57 (49–65)	57 (47–66)	57 (49–65)
Age at first CVD event; median (IQR) (in years)	52 (45–60)	53 (45–63)	53 (45–61)
Duration since first CVD diagnosis; median (IQR) (in years)	2 (1–5)	2 (1–6)	2 (1–6)
Marital status; *n* (%)			
Not married	5 (2.0)	7 (4.9)	12 (3)
Married	227 (90.1)	97 (67.8)	324 (82)
Divorced/widowed	20 (7.9)	39 (27.3)	59 (14.9)
Education group; *n* (%)			
High	104 (41.3)	47 (32.9)	151 (38.2)
Middle	114 (45.2)	69 (48.3)	183 (46.3)
Low	34 (13.5)	27 (18.9)	61 (15.4)
Occupation status; *n* (%)			
Employed	166 (65.9)	41 (28.7)	207 (52.4)
Unemployed	30 (11.9)	80 (55.9)	110 (27.8)
Retired	56 (22.2)	22 (15.4)	78 (19.7)
Family history of CVD; *n* (%)	64 (25.4)	31 (21.7)	95 (24.1)
Diabetes; *n* (%)	47 (18.7)	22 (15.4)	69 (17.5)
Self‐reported smoking status; *n* (%)	22 (8.7)	0 (0)	22 (5.6)
Medication prescribed			
Antihypertensive drugs	223 (88.5)	122 (85.3)	345 (87.3)
Lipid‐lowering drugs	181 (71.8)	95 (66.4)	276 (69.9)
Antithrombotic agents	197 (78.2)	106 (74.1)	303 (76.7)
Blood parameters; median (IQR)			
HDL‐C (mmol/L)	1.2 (1–1.3)	1.3 (1–1.5)	1.2 (1–1.4)
Total‐C (mmol/L)	4.7 (3.9–5.4)	4.9 (4.1–5.9)	4.7 (4–5.6)
LDL‐C (mmol/L)	2.7 (2.3–3.5)	2.9 (2.3–3.9)	2.8 (2.3–3.6)
FPG (mmol/L)	6.1 (5.5–7.3)	5.9 (5.3–7.1)	6.1 (5.4–7.2)
Triglycerides (mmol/L)	1.4 (1.2–2.1)	1.4 (1.1–2)	1.4 (1.1–2.1)
Creatinine	85.8 (72.1–106.1)	67.2 (57.4–88.4)	79.6 (63.7–101.7)
Physical examination; median (IQR)			
SBP (mmHg)	129 (117–141)	130 (120–150)	130 (117–146)
DBP (mmHg)	76 (70–83)	75 (69–80)	76 (70–82)
WC (cm)	89 (83–97)	85 (78–93)	87 (82–95)
BMI (kg/m^2^)	23.6 (22–25.7)	23.9 (21.3–26)	23.8 (21.9–25.8)

*Note:* Categorical variables are presented as absolute numbers with percentages, and continuous variables are presented as medians with IQRs.

Abbreviations: BMI = body mass index, CVD = cardiovascular disease, DBP = diastolic blood pressure, FPG = fasting plasma glucose, HDL‐C = high‐density lipoprotein cholesterol, IQR = interquartile range, LDL‐C = low‐density lipoprotein cholesterol, SBP = systolic blood pressure, Total‐C = total cholesterol, WC = waist circumference.

### 3.2 CVD Risk Factor Target Attainment

**Figure 1:**
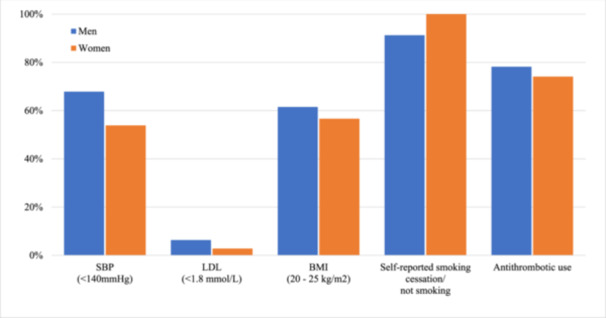
Distribution of CVD risk factor treatment target attainment stratified by sex. BMI = body mass index, LDL = low‐density lipoprotein cholesterol, SBP = systolic blood pressure.

Figure 1 illustrates the distribution of CVD risk factor target attainment stratified by sex. The median number of risk factor targets attained was 3 (IQR: 2–4). The lowest attainment rate was observed for the LDL‐C target, with 5.1% of patients reaching the recommended LDL‐C treatment goal. The attainment rates for BMI, SBP and antithrombotic use targets were 59.7%, 62.8% and 76.7%, respectively. Notably, most patients reported smoking or not smoking cessation, for a total of 94.4%. Overall, 1.8% of participants achieved complete attainment of every CVD risk factor target (men and women were 2.4% and 0.7%, respectively).

### 3.3 Risk Factor Attainment in Subgroups

**Figure 2:**
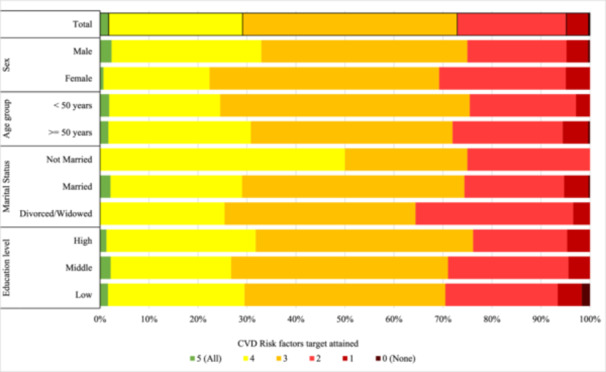
Composite number of risk factor treatment targets attained in established CAD patients.

A low attainment of CVD risk factor targets was observed across all patient subgroups to a similar extent (Figure 2). Multivariate logistic regression analysis (Table 2) revealed that the attainment of individual risk factor targets was stable across age, sex, marital status, and education level subgroups, except for blood pressure control, which was less often achieved in individuals older than 50 years (odds ratios [OR] 0.58, 95% CI: 0.36–0.94), and in women (OR 0.55, 95% CI: 0.36–0.84) adjusted for other characteristics.

**Table 2:** Multivariate analysis of CVD risk factor target attainment across patient subgroups.

	Attained risk factors treatment target (OR 95% CI)									
	SBP		LDL		BMI		Smoking status*		Antithrombotic use	
Patients' characteristics	Crude	Adjusted	Crude	Adjusted	Crude	Adjusted	Crude	Adjusted	Crude	Adjusted
Age group										
< 50 years	1 (ref)	1 (ref)	1 (ref)	1 (ref)	1 (ref)	1 (ref)	1 (ref)	1 (ref)	1 (ref)	1 (ref)
50+ years	**0.58 (0.36–0.94)**	**0.57 (0.35–0.94)**	0.85 (0.32–2.27)	0.87 (0.31–2.41)	1.19 (0.76–1.87)	1.29 (0.81–2.05)	1.02 (0.39–2.69)	0.84 (0.29–2.43)	1.54 (0.93–2.56)	1.58 (0.94–2.65)
Sex										
Men	1 (ref)	1 (ref)	1 (ref)	1 (ref)	1 (ref)	1 (ref)	1 (ref)	1 (ref)	1 (ref)	1 (ref)
Women	**0.55 (0.36–0.84)**	**0.53 (0.34–0.83)**	0.42 (0.14–1.30)	0.41 (0.13–1.29)	0.82 (0.54–1.24)	0.87 (0.56–1.36)	NA	NA	0.80 (0.50–1.29)	0.80 (0.48–1.32)
Marital status										
Not married	1 (ref)	1 (ref)	1 (ref)	1 (ref)	1 (ref)	1 (ref)	1 (ref)	1 (ref)	1 (ref)	1 (ref)
Married	0.58 (0.15–2.19)	0.55 (0.14–2.20)	0.61 (0.07–5.00)	0.62 (0.07–5.78)	0.51 (0.14–1.92)	0.43 (0.11–1.64)	3.21 (0.66–15.7)	6.69 (0.87–51.19)	0.65 (0.14–3.04)	0.53 (0.11–2.51)
Widowed/divorced	0.42 (0.10–1.72)	0.55 (0.13–2.37)	0.39 (0.03–4.64)	0.50 (0.04–6.83)	0.37 (0.09–1.50)	0.32 (0.08–1.34)	11.6 (0.96–140.26)	12.24 (0.73–205.66)	0.64 (0.13–3.29)	0.53 (0.10–2.76)
Education level										
High	1 (ref)	1 (ref)	1 (ref)	1 (ref)	1 (ref)	1 (ref)	1 (ref)	1 (ref)	1 (ref)	1 (ref)
Middle	0.72 (0.46–1.12)	0.74 (0.47–1.17)	1.25 (0.43–3.59)	1.32 (0.46–3.81)	1.01 (0.65–1.57)	1.04 (0.66–1.62)	0.59 (0.22–1.61)	0.55 (0.20–1.53)	1.01 (0.61–1.69)	1.03 (0.61–1.73)
Low	1.05 (0.56–1.97)	1.16 (0.61–2.22)	2.16 (0.63–7.35)	2.37 (0.68–8.29)	0.60 (0.33–1.10)	0.61 (0.33–1.12)	0.59 (0.16–2.17)	0.59 (0.14–2.46)	0.93 (0.46–1.85)	0.93 (0.46–1.87)

*Note:* The odds ratio (OR) was adjusted for all variables in the column; an OR < 1 indicates a lower probability of attaining the risk factor treatment target in comparison to the reference group. (*) Self‐reported smoking status; a bold value indicates statistical significance (*p* < 0.05). The risk factor control target defined as follows: SBP < 140 mmHg; LDL‐C < 1.8 mmol/L; BMI = 20–25 kg/m^2^; smoking status = self‐reported smoking cessation or not smoking; and antithrombotic use = the use of anticoagulants, antiplatelet agents or their combination.

Abbreviations: BMI = body mass index, CI = confidence intervals, LDL‐C = low‐density lipoprotein cholesterol, NA = not applicable, SBP = systolic blood pressure.

We were unable to analyze the association between patient sex and self‐reported smoking status because all female participants reported being nonsmokers or had quit smoking.

## 4 Discussion

This study demonstrated an alarmingly low rate of attainment of risk factor treatment targets, except for smoking, based on the current Indonesian ASCVD prevention guidelines. Only 1.8% of the patients were able to achieve the recommended levels for all the risk factors studied. Among these risk factors, LDL‐C had the lowest attainment rate, indicating a significant challenge in achieving optimal cholesterol control. Moreover, a similar distribution of patients with low attainment of risk factor treatment targets occurred in all subgroups.

### 4.1 Systolic Blood Pressure

Our study showed that the nonattainment of recommended SBP levels in this population remains high, with 37% of participants not achieving the SBP target. This is lower than that reported in other studies conducted in various regions of the world, including Asia, where a significant proportion (46%–65%) of patients with established CAD failed to reach the recommended blood pressure target [14]. Globally, challenges in attaining SBP targets are often linked to high levels of salt intake, insufficient physical activity, and obesity, as seen in several developed countries. In contrast, local factors, including regional inequalities, socio‐demographic characteristics, health literacy, cultural practices, and governance structures, significantly influence the attainment of health targets in Indonesia [15, 16, 17, 18]. Moreover, our results showed better results than those of a nationwide survey from Indonesia in 2018, in which more than half (59.6%) of CVD patients had blood pressure measurements above the recommended level [19].

In the present study, we observed that female patients had lower rates of achieving the recommended target for SBP, while in developed countries, both genders achieved similar SBP targets post‐treatment [20, 21, 22]. Interestingly, despite better hypertension awareness, medication adherence, and regular blood pressure monitoring than in male patients, according to the findings from the Indonesia Family Life Survey [23], additional female‐specific factors such as menopause, oral contraceptives, pre‐eclampsia, and other biological factors outweigh the low attainment of SBP treatment targets among females in our population. This gender‐related challenge is observed globally but may have amplified effects in Indonesia due to societal norms and healthcare practices that differ from those in developed countries. Women with CVD face unique social and psychological challenges due to cultural expectations, highlighting the need for culturally sensitive healthcare approaches [24, 25].

In Indonesia, the control of blood pressure encounters significant challenges due to various patient‐related barriers [26], including the discontinuation of blood pressure‐lowering medications after a period of perceived wellness or after improvement in symptoms. Furthermore, other hurdles to overcome include limited access to healthcare facilities, reliance on herbal remedies or alternative treatments, forgetfulness or lack of awareness, and several other reasons [26].

### 4.2 Low‐Density Lipoprotein Cholesterol

Our study revealed that LDL‐C had the lowest achievement rate among CAD patients across all subgroups. Consistent with our findings, a study conducted by Poh et al. in 2018 on nine Asia‐Pacific countries, including the population with stable CAD from Indonesia, demonstrated that many of these individuals have elevated LDL‐C levels, and only a few were able to meet the recommended target according to the guidelines [27]. This trend is also observed across Europe, where only a small proportion of high‐risk patients achieve LDL‐C targets, with just 16% of patients with established CVD and 18% of those at very high risk reaching LDL‐C levels < 1.8 mmol/L. In specific countries, the attainment rates are similarly low, such as in Italy, where only 3.2% of very high‐risk patients with ASCVD meet the LDL‐C goals set by the 2019 ESC/EAS guidelines [28, 29]. In addition, our previous study in 2022 suggested that the limited availability of essential drugs, including statins, and inadequate capacity for comprehensive lipid profile testing to monitor lipid status at primary healthcare facilities in Indonesia may have contributed to the low attainment of LDL‐C treatment targets [30]. Another factor that may further contribute to the increased incidence of high LDL‐C levels in the Indonesian population is the tendency to consume foods with high amounts of saturated fat (one to six times per week) and maintain a relatively low intake of fruits and vegetables (one to two portions per day per week), even in individuals with established CVD [9, 19].

### 4.3 Body Mass Index

A significant proportion of patients had not attained the recommended BMI target, and the distribution was also similar across patient subgroups. Previous systematic reviews and meta‐analyses have consistently shown that elevated BMI is related to a similar risk of developing CAD in both men and women, regardless of age, ethnicity, or socioeconomic status [31]. This association has been also observed in diverse settings across various countries, such as the United Kingdom, Denmark, and the United States, where elevated risks of CHD are seen in both overweight and obese populations (pooled *p*‐value < 0.001). Another study reported that individuals with lower levels of formal education, particularly those with an elementary education or below, tend to have a greater likelihood of not attaining the recommended BMI [26].

### 4.4 Smoking Status

We observed a high attainment rate of not smoking or smoking cessation in this high‐risk population (< 5%). However, in contrast to the findings of a previous study (2022), approximately 64% of Indonesian adults were smokers, with men comprising the majority. Furthermore, approximately 20% of the population continues to smoke even after being diagnosed with CVD [19]. This differs from what has been observed in a study from South Korea, where individuals with CVD had higher smoking cessation rates compared to those without CVD, suggesting that a CVD diagnosis itself may motivate quitting [32]. Another study in 2018 showed a higher incidence of current smoking (25.8%) and former smoking (36.5%) in patients with acute and stable CAD [9].

In the present study, questionnaires regarding smoking behaviour were collected from patients who were previously hospitalized for MI or who underwent PCI. This may have introduced reporting bias, potentially leading to an underestimation of the actual smoking prevalence. This underestimation aligns with previous research on the accuracy of self‐reported smoking behaviour in clinical settings, especially in cardiac patients [33].

### 4.5 Use of Antithrombotic Agents

Our study revealed that 76% of CAD patients were receiving antithrombotic therapy at the time of observation, either as antiplatelet or anticoagulant medication. Notably, the distribution of antithrombotic agents used was similar between men and women, as was the distribution of other patient subgroups. A previous study investigating antithrombotic management patterns in Asian ACS patients revealed that 88.1% of postcardiac intervention patients received dual antiplatelet therapy (DAPT) within 12 months after hospital discharge. However, over 2 years, this percentage gradually declined, with only 61.5% of patients remaining on DAPT [34].

### 4.6 Challenge for Improving the Attainment of CVD Risk Factor Control Targets

Our study showed that CAD patients who did not achieve treatment targets were evenly distributed in all the subgroups. Therefore, prioritizing risk factor treatment and promoting therapy adherence are crucial for every subgroup in this high‐risk population. Evidence‐based and cost‐effective pharmacotherapies for achieving treatment targets in primary and secondary prevention should be made available in primary healthcare facilities [35]. However, essential drugs for controlling CVD risk factors, such as antihypertensive, lipid‐lowering and antithrombotic drugs, are underutilized [36] and are often unavailable in Indonesian public primary healthcare facilities [30].

Suboptimal secondary prevention of CVD in resource‐limited countries is influenced by various constraints across different aspects of the disease continuum, involving patient, clinician, and health system factors [37]. Patient‐related barriers include insufficient knowledge, attitudes, awareness, and treatment nonadherence, which are common [38]. Additionally, challenges related to socioeconomic factors, including poor education, low income, and unemployment, hinder access to quality healthcare and medication [39]. Moreover, clinicians in resource‐limited settings encounter several barriers that interfere with the optimal secondary prevention of CVD. These barriers include a lack of awareness of the latest guidelines and evidence‐based interventions for CVD management, time constraints, and inadequate resources to implement evidence‐based therapies [40, 41, 42]. Addressing these issues requires ongoing education and training for clinicians, as well as the adequate allocation of resources to support comprehensive CVD care [8]. Furthermore, at the health system level, some barriers include fragmented care, low prioritization of prevention, and inadequate infrastructure for systematic screening and management of CVD treatments, as indicated by previous studies [43, 44, 45].

These aforementioned constraints collectively contribute to the challenges faced in achieving optimal secondary prevention of CVD, especially in resource‐limited countries. Establishing a CVD registry to continuously provide valuable insights into care practices and achieving treatment targets in high‐risk patients could help address this issue. The registry could assess current clinical practices and monitor patients' conditions post‐hospital discharge [46].

### 4.7 Strengths and Limitations

The present study employed a consecutive sampling approach to select individuals with established CAD in Indonesia. This approach minimized potential bias in participant selection and improved the representativeness of our findings. By including individuals at very high risk, we focused on identifying a group that potentially derives the greatest benefit from evidence‐based preventive interventions.

However, some limitations need to be acknowledged. First, the reliance on self‐reported smoking status may have led to an underestimation of the actual prevalence of smoking behaviour. A more objective observation could provide a more accurate picture of smoking behaviour in this high‐risk population. Second, we did not collect any information about the patient's long‐term treatment history, medication adherence, or whether they were enroled in any intervention study aimed to reduce their risk factor levels which could have provided valuable insights for better explaining our results.

Furthermore, it is important to note that our study employed a cross‐sectional design, which hindered the assessment of changes in risk factors over time. Additionally, as a single‐centre study, the generalizability of our findings to a larger population may be limited. Caution should be exercised when extrapolating these results to broader contexts. Future research should address these limitations to enhance our understanding of the topic.

## 5 Conclusion

The attainment of treatment targets for controlling CAD risk factors, as recommended in the CVD prevention guidelines, was found to be very low in our population. Less than 2% of the patients were able to achieve the recommended levels for all the risk factors studied. This trend was consistently observed across various subgroups. These findings emphasize the pressing need to enhance awareness among patients and healthcare providers about managing and controlling risk factors, especially concerning LDL‐C, within this high‐risk population. Prompt action is crucial for mitigating the significant burden of CVD, especially in low‐ and middle‐income countries such as Indonesia.

## Author Contributions

D.S.A., S.H.J.H. and M.J.C. were responsible for the conceptualization and design of the study. Data collection was conducted by D.S.A., W.M., A.Q. and N.Q. The data were analyzed by D.S.A., S.H.J.H. and A.Q. D.S.A., A.Q. and N.Q. drafted the manuscript. F.L.J.V., P.A.D., A., W. and I.M. supervised the study and contributed to the subsequent revision of the manuscript. All the authors read and approved the final manuscript.

## Disclosure

The funding body played no role in the design of the study, data analysis and interpretation of data or in writing the manuscript.

## Ethics Statement

The study was conducted in accordance with the Declaration of Helsinki and approved by the Institutional Review Board of the Faculty of Public Health, Hasanuddin University (approval document number: 8361/UN4.14.1/TP.02.02/2020). Informed consent was obtained from all participants involved in the study before the interviews.

## Conflicts of Interest

The authors declare no conflicts of interest.

## Data Availability

The data that support the findings of this study are available from the corresponding author upon reasonable request.
